# Laboratory-based diagnosis of scabies: a review of the current status

**DOI:** 10.1093/trstmh/trab049

**Published:** 2021-03-25

**Authors:** Emmanuel Edwar Siddig, Roderick Hay

**Affiliations:** Nile University, Faculty of Medicine, Khartoum, Sudan; Erasmus Medical Center, University Medical Center Rotterdam, Department of Medical Microbiology and Infectious Diseases, Wytemaweg 80 3015 CN, Rotterdam, The Netherlands; Kings College London, Guys Campus, London SE1 9RT, UK

**Keywords:** direct microscopy, histopathology, LAMP, molecular diagnosis, scabies, serology

## Abstract

Scabies is a neglected tropical disease (NTD) of the skin that is caused by the mite *Sarcoptes scabiei*. It is considered to be an important public health problem in many regions. The disease is at its most prevalent in low-resource countries where there are overcrowded living conditions coupled with poor hygiene. In some regions, mass drug administration using ivermectin is a key population-based approach to the control of scabies. Before starting a patient on specific treatment, confirming the diagnosis by accurate and rapid identification of the organism is critical. Different laboratory-based techniques for scabies have been developed in the last few decades. These include direct microscopy and histopathology. More recently, serological testing, dermoscopy and different molecular techniques have been developed as diagnostic methods for scabies. To date, none of these, apart from microscopy and dermoscopy, has been translated into routine clinical laboratory practice. A simple point-of-care or laboratory test would provide a rapid and confirmed diagnosis and early institution of effective treatment. In this review we present an update on the laboratory techniques currently in use for the identification of scabies.

## Introduction

Scabies is a neglected tropical disease (NTD) of the skin that attracts scant health and research attention globally. It is a parasitic infestation that is caused by the ectoparasite *Sarcoptes scabiei*.^1–3^ Scabies mites of this genus infect animal species, where infection is known as mange, as well as humans, there being small but detectable molecular differences in the genomes of the variants or strains. In humans, scabies is considered to be a global health problem, as >130 million individuals are estimated to be affected, with a predominance of infections in tropical regions such as the West Pacific, sub-Saharan Africa and Latin America.^2^^,^^3^ Secondary bacterial infection caused by streptococci and *Staphylococcus aureus* leads to further morbidity through sepsis, glomerulonephritis and rheumatic fever. Accordingly, many countries have identified the disease as a public health problem that needs effective resolution and the World Health Organization (WHO) has included scabies as a formally designated NTD.^1–4^

The diagnosis of scabies is challenging and potentially time consuming and the disease can exhibit different clinical behaviours as well as responses to treatment.^5^^,^^6^ Furthermore, secondary bacterial infections may affect the clinical expression of the disease.^7–10^ Careful clinical history taking and examinations are needed for the diagnosis of scabies. To aid this, various clinical diagnostic schemes and algorithms have been devised. The latest is one recently developed by the International Alliance for the Control of Scabies (IACS), which has been adopted as a key diagnostic aid.^11^ However, new laboratory tests and techniques are also needed to identify the presence of the mite. In most clinical settings it is essential to make an accurate diagnosis before initiating treatment plans for patients or to support major public health interventions with mass drug administration using ivermectin. Presently there is no rapid, field-friendly, point-of-care test available for the identification of scabies. Using ink to highlight burrows provides a partial answer but does not distinguish old from new lesions. This complicates the diagnosis of scabies in rural, remote endemic regions with meagre medical and health facilities. In well-resourced health services the use of dermoscopy or skin surface microscopy is one way of identifying the presence of mites, but it is costly and requires training. One approach that has been applied to address this issue and reduce the cost is through training local health workers in the use of visual identification through video microscopy followed by extraction and identification of mites.^12^ However, a simple point-of-care test would be a more practical approach to diagnosis in field settings. Herein we discuss the different laboratory-based tests and techniques that have been applied to the diagnosis of scabies.

## Direct microscopy

Direct microscopy is considered to be a standard and reliable diagnostic technique for rapid diagnosis in which skin over the burrow of a scabies mite, identified by its appearance, is scraped or the contents removed on a sterile needle and the material sent to a laboratory. The sample is placed on a slide and 5 drops of 10% potassium hydroxide or normal saline are added. The sample is covered with a cover slip and visualized under a microscope for the presence of mites, larvae or ova.^13^^,^^14^ While in skilled hands this technique is invaluable, it requires training and the sampling techniques are prone to error. Rapid transport of intact samples to a laboratory is also difficult in remote or rural settings. This method is how the scabies mite was originally described in a patient in the late 17th century.

## Histopathological diagnosis of scabies

For histopathological diagnosis, the scraped tissue or skin biopsy is fixed immediately in 10% formal saline or formalin solution. Paraffin blocks are prepared and cut with a rotary microtome in 3- to 5-µm sections. The sections are then stained with haematoxylin and eosin (H&E) for identification.^15^

Histopathological diagnosis of scabies is considered to be one of the most accurate tests currently available. However, it is time consuming and may take 2–7 d to obtain a result using routine fixation processes. Also, as with direct microscopy, selecting the right site for sampling is critical and it is an invasive procedure, therefore histopathology is not considered as part of the routine workup for the diagnosis of scabies and is reserved for the confirmation of difficult or atypical cases.

**Figure 1.**
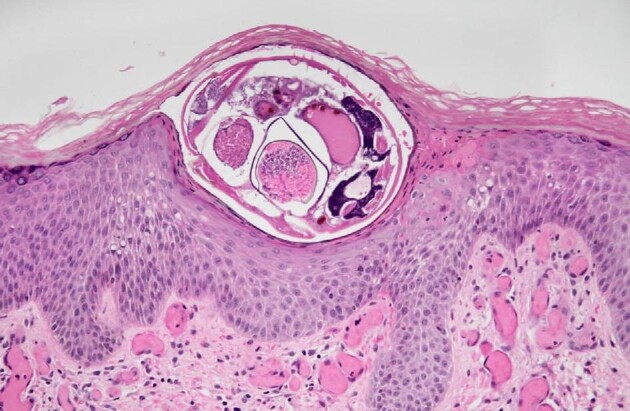
Skin biopsy showing scabies mites in the epidermis (H&E, ×40).

Histopathology reveals the mite parts, the immature forms and/or the scybala (faecal pellets) within tissue sections (Figure 1). The exoskeleton is thin and is coated with spines that can be visualized with H&E stain. Histopathological examination can be used to accurately diagnose the disease if the mite parts are present; however, where these cannot be visualized a polarizing microscope may aid the diagnosis by identification of the detached spines that are not detected by H&E staining alone.^17^ Other pathological changes that involve the dermal and epidermal layers may be found with scabies. These include perivascular and interstitial inflammatory infiltration of the dermis, hyperkeratosis, acanthosis, epidermal spongiosis, acantholysis, vasculitis and the formation of superficial fibrin thrombi.^16^

## Serology

Several serological assays for scabies have been developed over the past decades. However, assays that have used whole mite extracts are time consuming and very expensive because they rely on a source of antigen, large numbers of the mites from a suitable host and there is no known culture medium for cultivation or method of growth amplification for *S. scabiei* var. *hominis*. Antigen extraction depends on the removal of infected material containing sufficient numbers of mites from human cases, such as those with crusted scabies, or the use of non-human mites from experimentally infested animals. Additionally, when using the whole extract, cross reactions occur with other mite species, including house dust mites such as *Dermatophagoides*, to which patients may be sensitised. To circumvent these problems, in 2007 Casais et al.^18^ developed an enzyme-linked immunosorbent assay (ELISA) based on identification of a 642 amino acid polypeptide using a recombinant *S. scabiei* var. *hominis* library. After insertion of the encoding complementary DNA in *Escherichia*  *coli*, antiserum was raised and used in Western blots of serum from chamois with scabies. The technique proved to be highly sensitive (100%) and specific (97%) in identifying infected animals. In 2010 Walton et al.^19^ developed a quantitative immunoglobulin E (IgE) inhibition assay for human use that identified IgE immunoreactivity of scabies mite antigens based on recombinant-produced *S. scabiei* cysteine or serine proteases and apolipoproteins. These antigens are located in the mite cuticle or gastrointestinal tract. They found significant IgE levels in patients with scabies compared with normal control subjects and, by using inhibition ELISA, no or minimal cross-reactivity with house dust mite antigens was observed. With the apolipoprotein Ssag 1.2, the sensitivity of the IgE assay was 88% and specificity was 100%. They tested patients with different clinical forms of scabies and found that greater IgE reactivity was seen for mite apolipoproteins with serum from patients with the crusted forms compared with normal scabies.

In 2015, Arlian et al.^20^ prepared aqueous extracts from *S.* scabiei var. canis as well as from common house dust mites. The antigen was then tested using sera from 91 patients and screened for IgA, IgE, IgG, IgM and IgD antibodies to *S. scabiei*. However, antibodies were found to cross react with house dust mites, including *Dermatophagoides farinae, Dermatophagoides pteronyssinus* and *Euroglyphus maynei*. Later, a further assay was developed with a recombinant *S. scabiei* actin-associated cofilin protein from rabbit scabies which is present in the splanchnic area, but not the exoskeleton, and shares 90% identity with *D. farinae* cofilin. One of the serological assays developed using this antigen of *S.*  *scabiei* was an indirect ELISA that was tested using rabbit serum from rabbits infected with scabies and uninfected controls as well as some with *Psoroptes cuniculi* and *Cysticercosis*  *pisiformis* infestations. This method showed 83.33% sensitivity and 87.9% specificity.^21^

A dissociation-enhanced lanthanide fluorescent immunoassay (DELFIA) was also developed based on a recombinant Sar s 14.3 major scabies antigen. No cross-reactivity was noticed using the house dust mite homologue Der p 14, and in a study of infested and control human subjects it proved to be a highly sensitive (100%) and specific (93.75%) method for scabies diagnosis in clinical settings.^22^

Another cloned protein that has been investigated is a *S. scabiei* tyrosine kinase (SsPTK), which is mainly located in the mouth part region of the scabies mite. It was evaluated in a rabbit model and was found to have a sensitivity of 95.2% and a specificity of 94.1%; it was also able to detect infection early in its course after 1 week.^23^ Other potential protein targets that have been assessed in experimental scabies models are triosephosphate isomerase,^24^ calmodulin^25^ and chitinase.^26^ In summary, detection of targeted scabies antibodies using immunoassays has shown promise, but the antigens targeted by these tests have not been adopted for use in immunologically based antigen detection assays.

## Molecular-based techniques

Scabies can also be identified by molecular methods that select different targets, including microsatellites, ITS-2 ribosomal DNA (rDNA), mitochondrial 12S/16S rRNA and S. scabiei myosin heavy chain genes (Table 1).^27–39^ Many of the published studies have focused on animal rather than human infestations. Additionally, an attempt was made recently to characterise the parasite proteins in order to establish the diagnosis using matrix-assisted laser desorption ionization–time of flight mass spectrometry (MALDI-TOF MS). The extracted and characterized proteins were not tested on clinical samples and the relevant databases had not been developed at the time of this study.^40^ However, this work has now generated details of many new molecular targets for subsequent proteomic work.

**Table 1.** Universal primers for the identification of scabies targeting different regions

Target	Primers	Annealing temperature, °C	Size (bp)	Reference
Unique site	Forward: 5′-ATTAAATCATTGCACAATAGAGCG-3′	60	138, 178	27
	Reverse: 5′-CTCATTAATTTTTTCCACCCTC-3′			
*cox1* gene	scab1 forward: 5′-CTTATTATTCCTGGATTTGGRTA-3′	55	250	35
	scab2 reverse: 5′CTAATTTTCCTCCTAATATTGTWGA-3′			
Mitochondrial 16S rDNA	SSUD forward: 5′-GGGTCTTTTTGTCTTGGAATAAA-3′	53	135	36
	SSUD reverse: 5′-CTAAGGTAGCGAAATCATTAGC-3′			
ITS-2 region	Forward: 5′-CTTTTTGAATGAATTTGC-3′	54	400	37
	Reverse: 5′-GTCATTTCTCGACCTCAGAT-3′			
	Probe: 5′-AAAGCACATCGATGGTGCGA-3′			

### DNA isolation

*S* *.*  *scabiei* DNA can be extracted using several different protocols. This can be accomplished directly from human samples such as skin biopsies^27^ and swab specimens.^35^ DNA can be extracted with commercial kits such as the Qiagen Tissue Kit (Qiagen, Hilden, Germany),^34^ QIAamp DNA minikit (Qiagen),^35^ Nucleospin Tissue kit (Macherey-Nagel, Düren, Germany)^36^ and silica magnetic NucliSENS easyMAG kit (bioMérieux, Marcy-l’Étoile, France).^37^

### Conventional polymerase chain reaction (PCR)

In 2001, Bezold et al.^27^ were able to identify S. scabiei in skin samples of a patient using primer sequences from highly conserved regions of *S. scabiei* microsatellite 15 (Sarms15). Later, in 2015, a further *S. scabiei*–specific conventional PCR was developed.^35^ This PCR was based on the mitochondrial cytochrome c oxidase subunit 1 (*cox1*) gene of *S. scabiei*, in which coding regions were selected that had no homology to any known sequences of potentially cross-reactive mites, including *Demodex folliculorum*, *Demodex brevis*, *Dermatophagoides pteronyssinus*, *Dermatophagoides farina*, *Dermanyssus gallinae*, *Tyrophagus putrescentiae* and *Cheyletus malaccensis*. PCR primers scabF1 and scabR2 appeared to be specific for *S. scabiei* and generated a 250 bp product. The sensitivity and specificity were 100%.^35^ The assay detected all 17 microscopy-positive patients and confirmed the diagnosis in an additional 12 patients. All these additional patients had compatible skin lesions and responded to antiscabetics.

In further work, another conventional PCR was developed by Angelone-Alasaad et al.^36^ in 2015 for the rapid diagnosis of scabies. Based on mitochondrial 16S rDNA, a primer set was developed that generated a PCR product of 135 bp with *S. scabiei.* This was used for the diagnosis of animal scabies, again using skin samples taken from six different animal species with confirmed sarcoptic mange (scabies). No amplification was obtained with other parasitic mites such as Psoroptes cuniculi and Notoedres cati var. and Otodectes cynotis.

Recently Delaunay et al.^37^ developed a PCR assay for identification based on the ITS-2 region that showed somewhat lower sensitivity to previously developed PCR assays in human scabies using a different sampling technique of swabbing the skin over the wrist and interdigital spaces of infested patients. Of 87 patients with dermatoscopically confirmed scabies, 33 had positive scabies PCRs, with a sensitivity of 37.9% and a negative predictive value of 61.7%. The authors pointed out the potential use of this as a screening method in scabies outbreaks.

### Real-time PCR

To discontinue the use of gel electrophoresis and to generate results more quickly for some causative agents, real-time PCR is appropriate.^35^ In 2015 a novel quantitative PCR (qPCR) targeting a 121-bp fragment of the *cox1* gene of *S. scabiei* was designed and evaluated using samples collected from human skin at different body sites before and after medical treatment. The newly developed qPCR could also be used to monitor treatment response, as the number of *S. scabiei* DNA copies was higher before the treatment and decreased after initiating treatment, becoming undetectable at days 14, 21 and 28 after the start of treatment.^35^ The authors also used swab sampling for one patient with crusted scabies and pointed out its simplicity as a sampling technique.

As a further approach to rapid detection, a TaqMan real-time PCR assay was developed in 2015 by Angelone-Alasaad et al.^36^ They used the assay as a diagnostic method for sarcoptic mange in different animal species. In this assay, a specific probe for *S. scabiei* was developed using amplification of 135 bp from mitochondrial 16S rDNA. The technique was highly sensitive and no cross-reactivity was observed. It was also more sensitive than endpoint PCR, as a minimum amount of *Sarcoptes* genomic DNA of 10 pg/µL was needed compared with 80 pg/µL for the conventional assay.

In 2020, Bae et al.^11^ developed an in-house reverse transcription PCR assay based on the *cox1* gene of *S. scabiei*, a 196 fragment using primers cox1F and cox1R with a cox1P2 probe. The authors used the IACS criteria as their case validation method. A total of 47 patients were tested; 33 had a suspected diagnosis of scabies, 10 had unrelated disease and 4 were healthy individuals. Of the 33 suspected cases, 22 had microscopy-proven scabies, 2 had clinically diagnosed scabies, 6 had suspected scabies and 3 were negative. Samples were obtained by scraping lesional skin. The assay showed a sensitivity of 86% in confirmed scabies cases, 83% in confirmed but clinically diagnosed scabies and 80% in clinically suspected scabies and 100% specificity.^38^ The results also matched the declining certainty of the diagnosis based on the IACS clinical criteria.

### Isothermal amplification techniques

The downside of using PCR-based identification tools is that there is still the need to use thermocyclers to amplify the DNA. In the past few years, isothermal amplification techniques have been developed to overcome this shortcoming. These include loop-mediated isothermal amplification (LAMP), rolling circle amplification (RCA) and multiple displacement amplification (MDA). These techniques are available to identify wide varieties of microorganisms. A LAMP assay has been designed based on the *ITS-2* gene for the identification of *S. scabiei* and has been found to be promising after evaluation of skin scrapings from infected animals with sarcoptic mange that showed 100% sensitivity and 92.3% specificity (Table 2).^39^

**Table 2.** Primer set for currently developed scabies LAMP

Pathogen	Target	Primer	Reference
*S. scabiei*	ITS-2 region	F3 TGTTAGTAGTAGCTCTATGAGAA	39
		B3 TCGCTTGATCTGAGGTCG	
		FIP ACCCTAGGAGAATGTCGCACAATGTTTCAAGTCTCGAGTGG	
		BIP CAGTGATGTGTGCCTGTTGAGAGAAATGACATTTCATTGCTTGT	
		LF CATCGATGTGCTTTCAA	
		LB CATGAATATCAAAGAGTG	

## Conclusion

As mentioned previously, using clinical examination alone may result in misidentification of cases, although the introduction of a validated clinical diagnostic scheme, the IACS diagnostic criteria, may improve this situation. In areas of high endemicity, mass drug administration to entire populations is appropriate, but in other settings, rapid diagnosis accessible from different healthcare setting remains of huge importance. Currently, molecular identification methods seem to be the most accurate and rapid way for diagnosing scabies. It is particularly important in areas of low endemicity, where mass treatment with ivermectin is not appropriate, or in atypical presentations of scabies.^41^ Several PCR methods for diagnosis have now been published and cited in this review. Most have been validated in clinical settings; however, ideally they should be tested for all forms of scabies (e.g. crusted forms) and in elderly patients. Also, some of these have been used for animal but not human scabies. The potential for small differences in genome sequences between human and animal variants of *S.*  *scabiei* to affect accuracy needs to be assessed in subsequent studies in humans.

However, in field settings, where simplicity and ease of access are crucial, efforts should be made to establish appropriate assays based not just on PCR, but also isothermal amplification techniques. Techniques that can be performed without the need for pure DNA are important, as DNA isolation in such settings can be the limiting factor. Once developed, these assays should be validated in multicentre clinical trials. Unfortunately, of the previously mentioned tools, none are currently suitable for field use and therefore will not be available for rapid diagnosis of the disease in low-resource settings. However, as new genomic data become available,^42^ the task of further refining tests should become simpler. Efforts should also be made to develop other point-of-care diagnostic tools that can be easily used for rapid and accurate diagnosis to ensure proper management of these patients.^41^

Detection of antibodies to specific antigen targets in scabies mites has also proved to be a potentially useful diagnostic tool, although, once again, more experience in human infections is needed. There is also the potential for cross-reactivity with common environmental mites. Methods for the detection of scabies antigens that can be adapted to clinic use have not been developed. Potentially these are easier to apply to point-of-care tests, providing the sampling methods, such as swabbing over affected sites, can be standardized and the sensitivity and specificity of the assay are suitable.

The adaptation and field testing of serological methods such as lateral flow assays and isothermal amplification molecular techniques or newer developments for use in peripheral health settings will improve the accurate diagnosis of this common infection.^43^ Tests based on these molecular methods have the advantage of sensitivity, as illustrated with the LAMP assay, but are more difficult to roll out for mass testing. Again, sampling methods in human populations will have to be standardised. Several target PCR probes as well as antigens and proteins have been identified, and adapting and evaluating their application to rapid diagnosis are now needed.

## Data Availability

None.
